# Pure Air and Pure Blood

**Published:** 1883-05

**Authors:** 


### PURE AIR AND PURE BLOOD.

DURING the early growth of the child, previous to birth, vessels are formed and a blood circulation established in them before a heart is developed capable of effective work. All the vegetable world and a majority of the animal creation are destitute of hearts, yet the fructifying juices flow freely upward, against the influence of the law of gravitation, from the roots to the topmost twig of the greatest trees. And the blood circulation seems to be as perfect in those animals that are destitute of hearts as in mammals, who are provided with a complicated cardiac apparatus.

The popular idea that the heart is the sole cause of the circulation is thus shown to be an error. The movements of the blood through the capillaries, of the contents of the lymphatics, and the motions of the muscular fluids are altogether independent of the heart's action. The chief function of this organ is to impart the initial impulse to the vital current, so that the arteries shall be always full, thus presenting an abundant supply of the vital fluid to the pulmonary and systemic capillaries, through which it flows mainly by the operation of a physical law discovered by the late Professor John W. Draper. He taught that this law consists in an attraction or affinity possessed by the blood for the sides of the vessels in which it moves. Notable examples of the operation of Dr. Draper's law may be cited. For instance, if a number of capillary glass tubes of different diameters be dipped into water, the latter will rise in the tubes above the level of the surrounding liquid ; the smaller the diameter of the tube, the higher will the water rise. The most familiar example of the operation of this law is observed in the ascent of coal oil in the wick of a lamp ; the fibres of which the wick is made form a series of capillary tubes ; the oil rises through them by the force of the affinity it has for the cotton, and it continues to flow upward in a steady stream as long as the oil is removed from the top of the wick by combustion.

The ascent of the sap of plants occurs by virtue of the same physical conditions ; it continues to ascend through the capillary tubes of which all plants are composed as long as the watery part escapes from the leaves. In the human blood circulation the following conditions obtain :

We have on one side the arteries containing blood rich in oxygen and the elements of nutrition ; on the other side, the venous blood, which has been despoiled of the vital gas and largely exhausted of nutrient properties ; between these lie the capillaries that receive the vital fluid from the former and deliver it to the latter. While the blood passes through the capillaries the exchange of nutritive material between it and the tissues occurs. Now, arterial blood containing oxygen with which it is ready to part must necessarily have a greater affinity for the capillaries than venous blood that has lost its oxygen, therefore the arterial blood enters the systemic capillaries, drives before it and expels on the other side of the capillary plexus the blood that has lost its affinity for the walls of the capillaries while traversing them. An important factor in the problem is the attraction of the tissues themselves for arterial blood ; this contains the material adapted to the nutrition of all the several tissues, the bony, the nervous, the muscular, etc.

Each several tissue has an elective affinity for the special matters adapted to its nourishment. Therefore blood containing these elements is powerfully attracted by the tissues requiring such nutriment, hence the circulation of the blood toward them. As soon as the required matters have been abstracted by the needy tissues from the blood it loses its affinity by parting with its nourishment and its oxygen, and by the absorption of carbonic acid gas and waste matters, and it is pressed on by the unchanged blood behind it. Therefore the motive power of the capillary circulation is chiefly the affinity possessed by the blood for the sides of the minute blood-vessels and the tissues which it is intended to nourish. But this affinity exists in a normal degree only in blood that is duly charged with oxygen introduced by adequate respiration. Dr. Draper says : "In my view of the subject it is, therefore, the arterialization of the blood in the lungs which is the cause of the circulation in man. I consider the circulation as the consequence of respiration, and though in one sense the minor causes are numerous, each portion of nervous material, each muscular fiber, every secreting cell working in its own way, these subordinate actions are all referable to the primordial act, and that is the exposure of the blood to the air."

Whatever, therefore, interferes with the respiration interferes with circulation. If an irrespirable gas be thrown into the cells of the lungs, the passage of the blood is instantly arrested and asphyxia ensues. Or, if the access of the air be cut off, as in drowning, in vain the heart exerts its utmost convulsive throb—it is unable to drive forward the blood. And in those cases by no means infrequent, yet undoubtedly the most surprising occurring in medical practice, restoration from death by drowning, the whole success turns on one condition, the re-establishment of the arterialization of the blood. If that be accomplished, the circulation is restored, and the heart proceeds with its duty.

The function of nutrition, considered in its widest sense, embraces the whole series of vital operations by which nutritive matters are digested, absorbed, vitalized, and appropriated by every part of the body, including those retrogressive changes through which the same materials are removed from the system after they have subserved the vital purposes.

The materials required for the sustenance of the body may all be included under three heads: air, water, and food. The Creator has beneficently adapted the supply of these necessaries to the urgency of the want. Breathing must continue without cessation, but what is as free as air? The necessity for water is not quite so imperative, and this liquid has been made almost as accessible as air. The demand for food is much less urgent, and to man has been said: "By the sweat of thy face shalt thou eat bread."

The amount of food required varies widely in different countries and among different races and individuals. A calculation made by the late Professor W. H. Draper, based on an examination of the diet scales of the English and French navies, led him to the conclusion that the average quantity of dry solid food required per day by a man in actual life was about two and a quarter pounds avoirdupois, or upward of 800 pounds per annum; he also estimated that about 1,500 pounds of liquid were taken, as water, tea, coffee, etc., and that the weight of oxygen entering the system by means of respiration was about equal to that of the food. The materials required for the sustenance of a man in the course of a year, therefore, exceed one ton and a half, or more than twenty times his own weight. All this vast amount is received into and assimilated by the body to maintain its integrity and to render possible the evolution of power, whether that be nervous, muscular or intellectual. After having done its duty, it is removed as waste matter.

These pregnant facts afford us a glimpse of the rapidity of the changes ever going on in the living body. Modern science has exposed the fallacies of the old physiologists, who taught that what they called the vital principle endowed the body with the power to resist change, and that this occurred only when life was extinct. The truth is that the living body submits to unceasing waste, which is the inevitable result of vital activity ; and if proper supplies of nutritive material be withheld, it soon perishes. Thus it is absolutely true that our bodies are ever dying.

The sole difference between this ever occurring interstitial death of atoms and final dissolution consists in the fact that, in the first instance, no sooner does one atom of a living body die than a living atom is supplied from the blood to take its place and perform its functions ; in second instance, the whole fabric returns to dust as it was.

The essential condition of physical life is the continual death of the atoms of which the body is composed, and their renewal by the operation of the nutritive process. Decay is, in fact, more truly a part of life than it is of death, because it goes on unceasingly during all our physical existence, but after dissolution it ceases when the work of decomposition has been completed. The living body is like the flame of a lamp, which may present the same aspect from hour to hour, but the incandescent particles of matter of which it is composed pass upward through it in a ceaseless stream. The body is like a noble mansion built of wonderfully wrought but perishable materials, the integrity of which is only preserved by repairs as ceaseless as the decay.

The rate at which the living body undergoes destructive change and renewal is much more rapid than is commonly supposed. It has been ascertained that a corpse will so resist decay, although no means have been taken to preserve it, that at the end of three and a half months the features will retain the aspect they wore during life, so that the individual may be recognized and the age told, while another month elapses before the features become unrecognizable and the large muscles of the body finally yield to decay.

On the other hand, experiments of too elaborate a character to be detailed here have proved that all the soft parts of a healthy human body are removed through destructive atomic change and completely renewed by the nutritive materials consumed as food in about three months and a half. Therefore, dead flesh and living flesh are equally perishable. What a significant corroboration by modern science on the words of the Book, "All flesh is grass, and the glory of it as the flower of the grass."

All the phenomena manifested by the living body arise by the mutual action and reaction of the air, water and the food on each other in the vital domain. These actions and reactions naturally resolve themselves into two distinct series : the progressive and the retrogressive. In the former the nutritive matters are digested, absorbed, vitalized, and appropriated by the living body as part and parcel of itself.

The absolute necessity of the oxygen received into the system by respiration to the carrying forward and completion of those wonderful processes by which the living body is built up and vitalized has justly earned for it the name of *the vital gas.*

The oxygen entrapped in the bubbles of saliva during the mastication of food and swallowed along with it enables the gastric juice to act more effectively on the food submitted to its action ; the solution of albuminoid food is thereby materially aided.

I have already stated that chyle gradually acquires the properties of blood while it is passing through the lymphatic system of tubes and the glands connected with it. Chyle corpuscles are developed there that eventually become red blood globules ; the following facts seem to be conclusive on these points :

Chyle drawn from the thoracic duct and exposed to the air coagulates like blood under the same conditions, and the clot becomes reddened in consequence of the absorption of oxygen by the chyle corpuscles from the air, showing that the immature blood is already competent to profit by the vitalizing influence of oxygen, to which its complete conversion into living blood while passing through the capillary vessels of the lungs is finally due.

Therefore, in every step of the nutritive processes from the beginning of digestion to the assimilation of the vitalized atoms into the living tissues, including the circulation of the blood, the oxygen introduced into the body by respiration is an essential element. Without this vital gas all these complex operations promptly cease, and if the quantity of oxygen be deficient, the vital processes and the material they elaborate for the nutrition of the body must be defective in a direct ratio with the meagreness of the supply.

After the living atoms have remained in the body a certain length of time, they are surrendered to the retrogressive or destructive series of changes, by which they are reduced step by step to the inorganic condition ; appearing finally as carbonic acid gas, which escapes by the lungs and the water, ammonia, sulphates, phosphates, etc., that are eliminated by the kidneys.

All the waste matters of the body arise therein by the oxidation of the food and the tissues, and without the influence of oxygen the reduction of the bodily débris to the gaseous and soluble forms by which their perfect elimination of the body is favored would be impossible. Therefore it is evident that, if the quantity of oxygen introduced into the body by breathing be inadequate, both the building up and the pulling down of the body and the disposal of the effete substances arising therein must be defective, causing in the first place various functional disorders, and, sooner or later, irremediable organic diseases and premature death.

The extreme importance of breathing is also shown by its urgency. Food and drink, especially the former, may be dispensed with for many days without destroying life, but if respiration be suspended for only a few moments the vital spark is soon extinct forever. The immediate effects of a total cessation of breathing are the accumulation of carbonic acid in the blood and a stagnation of the circulation, first in the capillaries of the lungs, and afterwards in those of the system generally, with an accumulation of blood in the venous system. As long as the heart continues to beat blood of a depraved quality and in steadily diminished quantity is sent to the nervous centres ; this blood exerts on them a depressing influence, so that consciousness is speedily extinguished, and the respiratory movements cease. The contractility of the heart is not finally lost, however, as soon as the breathing stops ; for some time after the left ventricle may be again set in motion by supplying it with arterial blood. Therefore, if the circulation has not been arrested too long, it may be renewed by artificial respiration. The entrance of fresh air into the cells of the lungs restores the circulation through the pulmonary capillaries, allows the venous blood to flow to the lungs ; this relieves the distension of the right side of the heart and conveys to the left the necessary stimulus to action. The whole vital apparatus is thus again set in motion.

The effects caused by the habitual breathing of a quantity of air but slightly less than the full requirements of the system are developed much more slowly, but they are in the highest degree insidious, and they produce in the end very much the same results—waste matters are imperfectly removed, the blood becomes impure and circulates sluggishly, the strength is reduced, and life is brought to a premature end directly or indirectly. Let us study these briefly in detail.

All the materials that can be used as food must be capable of uniting with oxygen. Oxidation is only another name for burning. All the waste matters arising in the body are fitted for removal therefrom by a chemical process identical with that which takes place in an ignited furnace. The combustion in the living body is very much less intense, but it is not the less genuine burning. In both cases it is the union of combustible matters with oxygen, and the chemical results are the same—heat is extricated and ashes remain.

The ashes of the living body are the matters that escape from the lungs, skin, kidneys, and bowels.

When the draught of the furnace is defective the coal is imperfectly consumed and the fire becomes choked with the débris. When the waste matters in the body are imperfectly oxidized, because the supply of oxygen to the system by breathing is too little, they cannot be totally eliminated unless they are perfectly burned. Under these circumstances they remain in the body to poison it, obstruct the vital operations, and to invite disease.

The human body in its career passes through certain determinate phases : it has an infancy, a youth, a maturity, decline, old age, and death. The same statements are true of the living particles of which it is composed ; they are also, so to speak, born, come to full maturity, and die. The atoms of which the body is composed must perish in order that the whole organism may enjoy a prolonged period of existence. The strength of the body is greatest during its maturity. So also is the vigor and vitality of the living atoms of which the body is composed greatest when they have attained full development.

When they are removed from the system as soon as their vitality begins to decline, and are replaced by young and vigorous living atoms, the whole body enjoys the highest degree of health and strength, in the same way that the effective strength of armies is preserved by retiring the fighting men before age has deprived them of youthful vigor, and replacing them with men in the prime of life.

During the process of training the skin of a pugilist becomes clearer, his muscles harder, his powers of endurance greatly increased, and his respiration deepened and lengthened. When fully trained he is able to endure fatigue and receive blows with comparative impunity that would have prostrated him before being submitted to the training.

All this notable improvement is attained by removing completely from his system all partially worn-out atoms and by replacing them quickly by new, vigorous, highly vitalized living particles.

When we consider that all the soft parts of the hnman body, as before stated, decay and are completely removed in about three months and a half, it is highly probable that under the rapid change of tissue induced by the severe training employed by pugilists the whole of their soft tissues are renewed in much less time.

All these changes are brought about by the union of oxygen with the decaying tissues, the retrograde change of the latter being hastened by active exercise. An adequate supply of the vital gas is essential to the process.

It is true exercise is an important factor in the renewal of the body, but the amount of exercise in which a man can indulge always bears a constant relation to the depth and freedom of his breathing. If the free entrance of fresh air into the lungs be obstructed, muscular exertion cannot be continued.

Therefore it is quite possible that a man who is well advanced in life, whose respiration is adequate to the full requirement of his system, may possess what is practically a young body; while a young man's system, because of inadequate respiration, may be so full of decaying and dead matter that for all practical purposes it is an old body.

We are told that "a little leaven leaveneth the whole lump;" this is due to the fact that a ferment possesses the power of reproductive growth when it is mixed with suitable material and kept at a proper temperature. A small quantity of yeast mixed with a large quantity of moist flour soon permeates the whole mass. The poisons that are believed to be the cause of all diseases that are communicated by contagion or infection are believed to act on the blood like a ferment. They are therefore called the zymotic diseases. Small-pox is a typical disease of this class. If a very minute portion of small-pox virus is placed beneath the skin of a healthy person, it will multiply in the course of a few days so that thousands of times the quantity of virus will be thrown out in pustules on his skin that was originally put into his blood. When the zymotic poisons that are believed to excite other acute diseases, such as typhoid fever, measles, or diphtheria, gain access to the body through the atmosphere, they are liable to produce the same effects as when introduced in a more tangible form, when that is possible.

Many facts have been accumulated showing conclusively that it is the absence or presence of waste matter in the blood of persons who are exposed to the malign influence of zymotic poisons that determines the susceptibility to their influence.

Of course it is an exceedingly important matter from a sanitary point of view to prevent the introduction of these poisons into the system; but there can be no question that the internal purity which is the uniform result of adequate respiration (other conditions being right) is the most effective safeguard against their deleterious action when exposure occurs, beause a ferment is only operative in the presence of fermentable materials, therefore, when a symotic poison finds access to the systems of persons whose blood is adequately purified by effective respiration, it is almost or quite incapable of causing disease except perhaps the dose be an overwhelming one, both because the waste matters necessary to its development are not present in appreciable quantity, and because under these circumstances the poison itself is quickly disposed of by the oxidizing process and eliminated from the system as readily as the waste matters normally arising in the system because of its functional activities.

All these facts justify the conclusion that adequate respiration promotes the internal purity of the body and directly contributes to health and longevity by reducing the tendency to the development of internal disorders, besides conferring greater immunity from diseases arising from external morbid influences.—*Dr. Work, in the Scientific American Supplement.*

